# Supplementary material for: Bacillus subtilis TO-A suppresses the age-related decline in locomotion in Caenorhabditis elegans
Source: Commun Biol. 2025 Oct 22;8:1492. doi: 10.1038/s42003-025-08879-y (PMC12546617; doi:10.1038/s42003-025-08879-y)
Supplement: Supplementary file 1 — Supplementary Information [file 42003_2025_8879_MOESM1_ESM.pdf]

Supplementary information

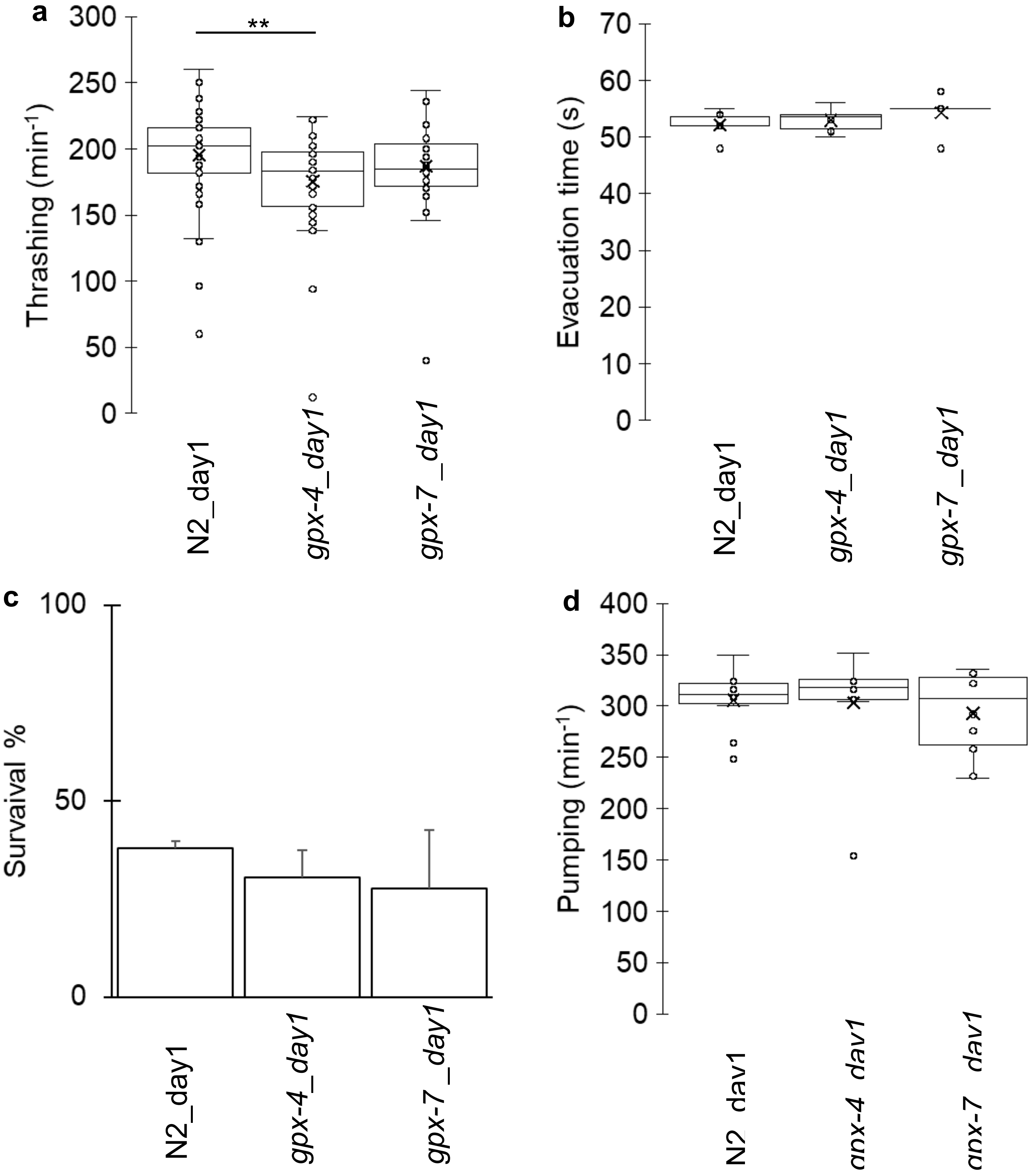

Supplementary Fig. 1. Locomotor function and oxidative stress resistance in young adults. (a) The thrashing rate, (b) evacuation time, (c) paraquat assay results, and (d) pumping frequency were measured using the day 1 adults of N2, *gpx-4* mutants, and *gpx-7* mutants. These experiments were performed at least twice independently. Error bars indicate the SEM..

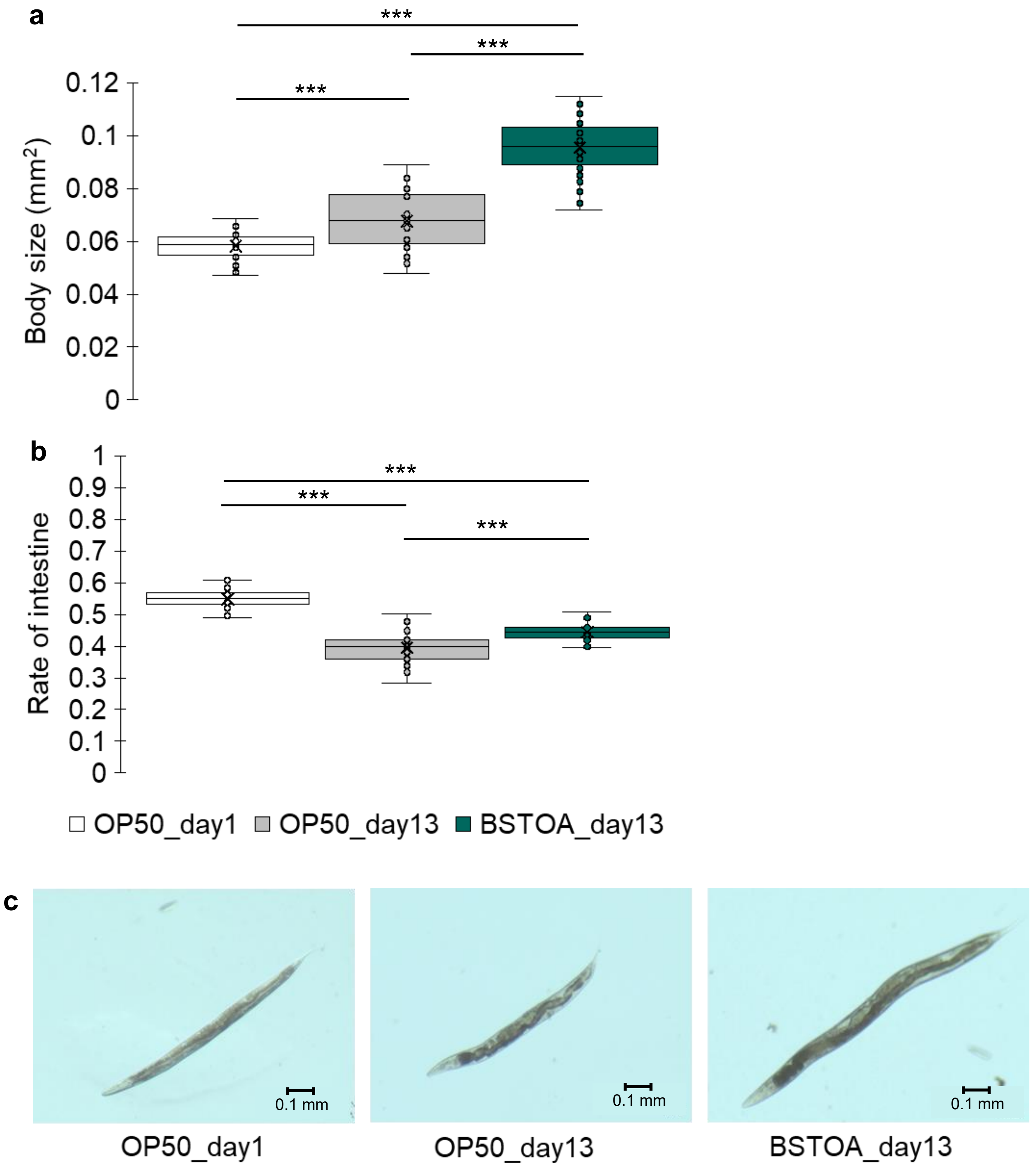

Supplementary Fig. 2. Body length and rate of intestine in the body of worms under each bacterial feeding condition. (a) Body length and (b) rate of intestine in the body of *C. elegans*. (OP50_day1: white; OP50_day13: gray; BSTOA_day13: green). (c) Representative micrographs of worms under different bacterial feeding conditions. Scale bar, 0.1 mm as indicated.

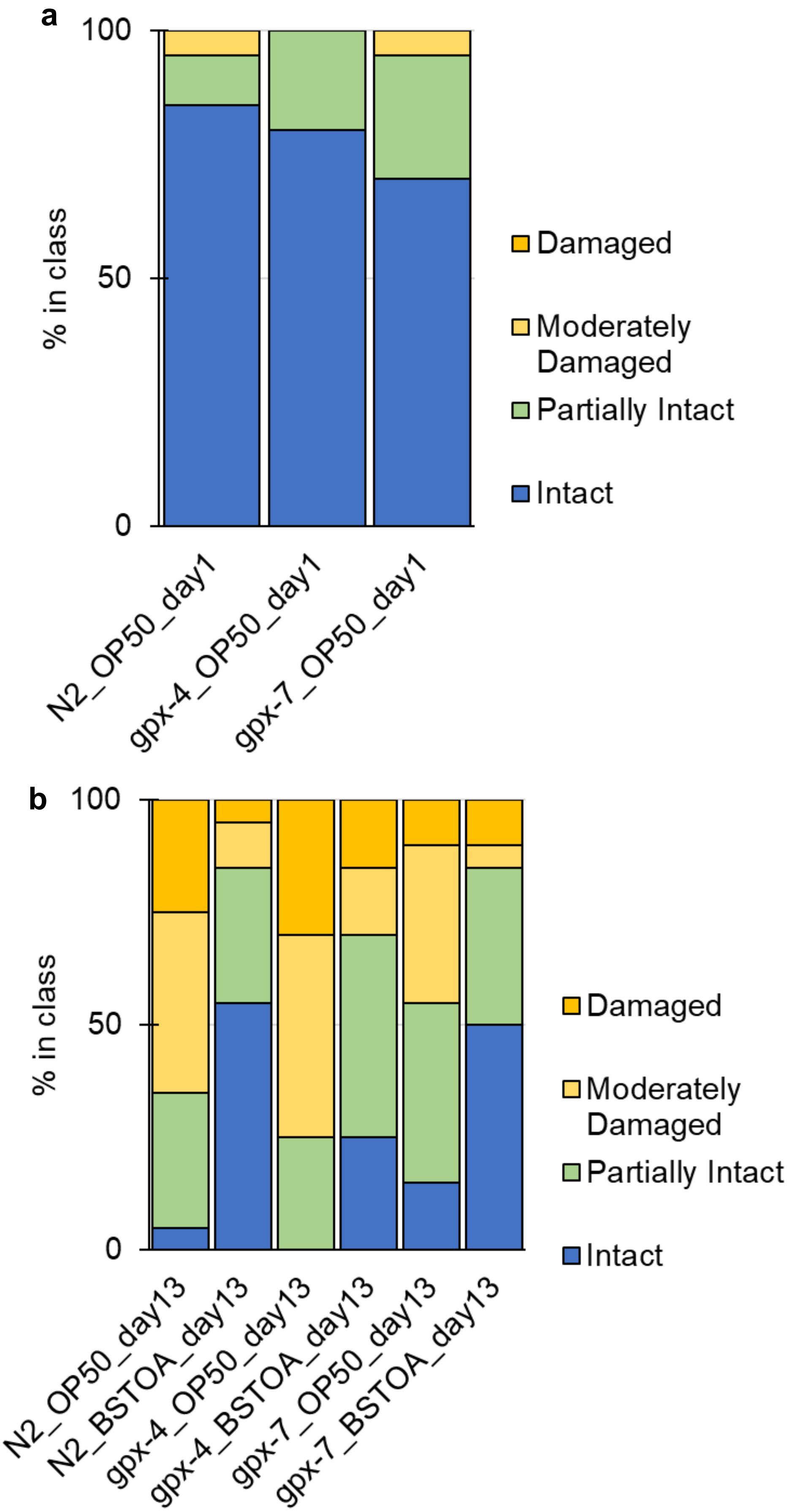
a
100
50
0
% in class
N2_OP50_day1
gpx-4_OP50_day1
gpx-7_OP50_day1
Damaged
Moderately Damaged
Partially Intact
Intact
b
100
50
0
% in class
N2_OP50_day13
N2_BSTOA_day13
gpx-4_OP50_day13
gpx-4_BSTOA_day13
gpx-7_OP50_day13
gpx-7_BSTOA_day13
Damaged
Moderately Damaged
Partially Intact
Intact

Supplementary Fig. 3. Status of body wall muscles in aged worms under each bacterial feeding condition. The evaluated status of the body wall muscles was classified into four distinct groups, intact, partially intact, moderately damaged, and damaged, using (a) day-1 adults of N2, *gpx-4* mutants, and *gpx-7* mutants and (b) day-13 adults of N2, *gpx-4* mutants, and *gpx-7* mutants fed OP50 or BSTOA. Each group consisted of 20 phalloidin-stained nematodes.

Supplementary Table 1. Sequences of oligonucleotides.

Gene Name	GeneID	Description	Forward primer (5′→3′)	Reverse primer (5′→3′)
*tba-1**	172831	Tubulin alpha chain	CGTACACTCCACTGATCTCTG	CTTTGGAACGACATCTCCTCTG
*cdc-42**	174233	Cell division control protein 42 homolog	CTATCGTATCCACAGACCGAC	CTGGATCATCCCTGAGATCG
*eif-3**	172858	Eukaryotic translation initiation factor 3 subunit C	TGCCACTGTTTCGCTCAAGAAG	GTGCATGATGAGACAGTCGGTG
*ama-1**	177190	DNA-directed RNA polymerase II subunit RPB1	CACAAAGTTTGGAGACCCGAGC	GGTTTGTTGGCTTGTGGAGTGATG
*pmp-3**	179968	ATP-binding cassette sub-family D member 4	CTACACTTTCACCGCCCAATGAC	TCCACATCCACTGTCTCCAGTTATC
T20H4.5	176023	putative NADH dehydrogenase [ubiquinone] iron-sulfur protein 8, mitochondrial	AGAATTCCGGCTCAACTTGCCG	CGTGACCAAGCATCACTCCGAA
sucg-1	177555	Succinate--CoA ligase [GDP-forming] subunit beta, mitochondrial	TGACGACAGTGCGGCTTATCGAC	GAGACCGGCTCCATTCACAAGA
acdh-13	177654	Acyl-CoA dehydrogenase family member 9, mitochondrial	CCGATTGTGACGCAAGGTGTAT	TCCAGAGCCTCCGTATTCTTCAG
clpp-1	174594	ATP-dependent Clp protease proteolytic subunit 1, mitochondrial	ATTAACAGTCCAGGCGGCAGTG	CTCGGCTGGTGAACCATGATTC
mrpl-15	171710	Large ribosomal subunit protein uL15m	AGATGTCTCAAGCTGCCCGTAG	TCCAGTGCTCCTTGTGTGTGAC
gst-4	177886	Glutathione S-transferase 4	CAAAGCTGAAGCCAACGACTCC	CAGCGTAAGCTTCTTCCTCTGC
gst-5	187537	GST C-terminal domain-containing protein;putative glutathione S-transferase 5	CGCTGGAGAAGTGTCTCGTCAG	TGGAGCAGCGCAGGTTTCTTTC
gst-38	185299	Glutathione transferase	CACGCGATGGCTCGTTATCTTG	CAAGCTTCACAGCGAGGTATGG
cep-1	172616	Transcription factor cep-1	ACGTTGTCGCAAGTTGTGCTC	TCGTTCTCTGGCACGCTTCTC

Gene Name	GeneID	Description	Forward primer (5′→3′)	Reverse primer (5′→3′)
asm-3	176879	Putative sphingomyelin phosphodiesterase asm-3;Sphingomyelin phosphodiesterase	AAGTTGTTCCGCCAGGCAAC	TGCGGAGTTCCACAGTCTGC
ubq-1	175840	Ubiquitin-related	GGCCAAGATTCAAGACAAGGAGG	AATCAGAGAGTGTGCGTCCATC
rac-2	186939	Ras-related protein rac-2	GTAAGACGTGTCTCCTGCTATCG	GATCGTAATCGTCCTGTCCAGC
ifb-2	173973	Intermediate filament protein ifb-2	ACAGAACCACCACAACTACATCC	AGGCAACGAACTCCTCAGC
daf-2	175410	Insulin-like receptor subunit beta;Protein kinase domain-containing protein;receptor protein-tyrosine kinase	CGGTGCGAAGAGAGGATATT	TACAGAGGTCGCCGTTACTG
daf-16	172981	Forkhead box protein O	GCCAAGCACTAACTTCAAGC	TTCCAGGCAGTGGAGATGAG
age-1	174762	Phosphatidylinositol 3-kinase age-1	TGGAGTTGCCTGAGCTGTC	GCGTGGAAGAGCCAATTCG
pmk-1	191743	Mitogen-activated protein kinase pmk-1	ATCATATACTTCATCCGACTCCAC	TACATTCAGCAGCACAAACAG
nsy-1	24104671	Mitogen-activated protein kinase kinase kinase nsy-1;mitogen-activated protein kinase kinase kinase	ACGGAACTCGTAGTGACTG	AGAAGTGGTAGAGAGCAAGG
atf-7	175587	Transcription factor atf-7	GAAATAAGGCTGCGGCTGTG	TACCCTTTCGAGTTCCCGTC
skn-1	177343	BZIP domain-containing protein;Protein skinhead-1	GCTACTCAATCGTTGTTCGATCC	CTGACGTGTATCGTGGAGATTC
mig-2	181344	Ras-related C3 botulinum toxin substrate 1;Signal recognition particle protein	TCCCAAAAGGCTCTTCTTCCC	GGATGCCATATAACGCTGACG
eps-8	178411	SAM domain-containing protein;SH3 domain-containing protein	AATGATGCGTCGAGGTGGATCG	ATTGGTTGTGACATTGGCACTCC

Gene Name	GeneID	Description	Forward primer (5′→3′)	Reverse primer (5′→3′)
unc-9	181443	Innexin;Innexin unc-9	CAGTGGTGCCAAGCAGGCAG	CGTAACAGGGTAGTTCTCCGG
unc-54	259839	Myosin-4	AGAAGCAGGTTGAGGAAGCTGAG	TTCAAGTTGGTGGGTGAGTTGC
set-18	172949	Histone-lysine N-methyltransferase set-18	CCAAAACAGGAGGATCCATTGG	GAGTGGCGAACGGTGTCTCG
ctl-1	259738	Catalase-2	GGCAAGATGGTGCTGAACAGG	GTAATGCGTGTCCGTGTAGGAG
ctl-2	175085	Peroxisomal catalase 1	AAGATGGTGCTGAACAGAAATCC	CGGTGTACGAGAAGATACGTCC
ctl-3	175086	Catalase	CATTTGATGTCACTAAAGTTTGGC	CGAGAACTCGATTCCTGGGAC
sod-1	174141	Superoxide dismutase [Cu-Zn]	TTCTCACTCAGGTCTCCAACGC	CTCCTTCAATAACTGCCTGGTC
sod-2	172632	Superoxide dismutase [Mn] 1, mitochondrial	AAAGGACGGAGGAGAACCATCG	CCCATCCTGATCCTTGAACAGC
sod-3	181748	Superoxide dismutase [Mn] 2, mitochondrial	GATGGTGGAGAACCTTCAAAGG	GTCTTTCTTGCAATATCCCAACC
gpx-1	184981	Glutathione peroxidase 1	TGAACGTCGCTTCACAGTGTGG	CGATTTCACACGATGGTTCCTG
gpx-2	187630	peroxidase;Glutathione peroxidase 2	AGGAGAAGACACTCCACTCTCC	GAATGCGAGAACTTCGAGTCCG
gpx-3	182513	Glutathione peroxidase 3	GAACGGAGACAATCATCACCC	GGAACTTCTCAAAGTTCCATGTG
gpx-4	190801	Glutathione peroxidase	CTCTGCGCTGGAAACAATGTGC	TCGGTGTACTGTTGAGTGTAGG
gpx-5	181178	Glutathione peroxidase	AGTCAATACCGTGGACAGGTGC	ATTCATCAGTTCATGGTTCTCAGC
gpx-6	188313	Glutathione peroxidase	CGAGTTGCCGCGTTTCCATGC	CAAAGTGGATGAGTGTTCTGACC
gpx-7	187542	Glutathione peroxidase	ATAGTGAATGTTGCATCATACTGC	GGTTCTTCTTCTCCAATCAATGG
gcs-1	174438	Glutamate--cysteine ligase	GGATTCCCAGGTCTCATTTCAC	GCAGGATGAGATTGTACGAATCC

*: Indicates the housekeeping gene

SUPPLEMENTARY MOVIE LEGENDS

Supplementary Movie 1.

Example video of (a) the pharyngeal pumping and (b) the locomotion behavior (Fig. 1b, d). These are used for pumping quantification and calculating moving velocity, as described in Methods.
